# Discovering context-specific relationships from biological literature by using multi-level context terms

**DOI:** 10.1186/1472-6947-12-S1-S1

**Published:** 2012-04-30

**Authors:** Sejoon Lee, Jaejoon Choi, Kyunghyun Park, Min Song, Doheon Lee

**Affiliations:** 1 Bio and Brain Engineering Department, KAIST, Daejeon 305-701, South Korea; 2 Department of Library and Information Science, Yonsei University, Seoul 120-749, South Korea

## Abstract

**Background:**

The Swanson's ABC model is powerful to infer hidden relationships buried in biological literature. However, the model is inadequate to infer relations with context information. In addition, the model generates a very large amount of candidates from biological text, and it is a semi-automatic, labor-intensive technique requiring human expert's manual input. To tackle these problems, we incorporate context terms to infer relations between AB interactions and BC interactions.

**Methods:**

We propose 3 steps to discover meaningful hidden relationships between drugs and diseases: 1) multi-level (gene, drug, disease, symptom) entity recognition, 2) interaction extraction (drug-gene, gene-disease) from literature, 3) context vector based similarity score calculation. Subsequently, we evaluate our hypothesis with the datasets of the "Alzheimer's disease" related 77,711 PubMed abstracts. As golden standards, PharmGKB and CTD databases are used. Evaluation is conducted in 2 ways: first, comparing precision of the proposed method and the previous method and second, analysing top 10 ranked results to examine whether highly ranked interactions are truly meaningful or not.

**Results:**

The results indicate that context-based relation inference achieved better precision than the previous ABC model approach. The literature analysis also shows that interactions inferred by the context-based approach are more meaningful than interactions by the previous ABC model.

**Conclusions:**

We propose a novel interaction inference technique that incorporates context term vectors into the ABC model to discover meaningful hidden relationships. By utilizing multi-level context terms, our model shows better performance than the previous ABC model.

## Background

With the advent of high-throughput methods and sheer volume of medical publications covering various diseases, biomedical researchers face challenges of distilling an enormous amount of data and discovering knowledge buried in them. Biological entities and their relations such as genes, proteins, molecules, processes, diseases, drugs and chemicals constitute underlying knowledge repository, and those entities and relations exist at various levels of entity types ranging from molecular to phenomic.

**Figure 1:**
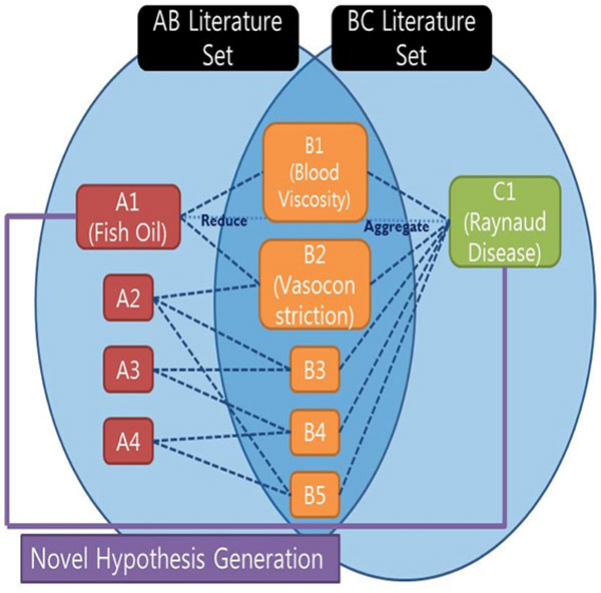
**Example of Swanson's UPK model**.

Discovering hidden relations among biomedical entities was first proposed by Swanson [1]. Swanson's Undiscovered Public Knowledge (UPK) model (a.k.a. ABC model) was to discover the implicit relations among biological entities such as magnesium, epilepsy, and migraine. As defined by Swanson, the ABC model is used for undiscovered knowledge which can be inferred by considering two (or more) complementary public relations [2] (see Figure 1). Discovering hidden relations is a daunting challenge specifically when multiple entities and relationships are interconnected at different levels. According to his ABC model, even though there is no connection reported between the concept A and the concept C, if there exists public associations between A and B, and between B and C, it is possible to infer a new relation between A and C. From this method, Swanson generated several hypotheses like "Fish oil can be used for treatment of Raynaud's Disease." Three years later, this hypothesis was proved clinically by DiGiacomo [3].

Several techniques have been developed to explore the Swanson’s ABC model. Weeber [4] attempted to discover novel relationships between drugs and diseases in the biomedical literature. With the ABC model, they developed the concept-based system by mapping words to UMLS concepts, and used it for Swanson’s Raynaud-Fish Oil and Migraine-Magnesium discoveries. Weeber [5] adopted the following two models to generate new hypotheses in discovering two processes: 1) an open discovery procedure with directional process and 2) a closed discovery procedure with bi-directional process.

Several studies employed the MeSH terms to infer the relationships between the biological objects [6-8]. Sehgal [6] explored genes and their relationships by using MeSH terms. Srinivasan [7] used MeSH terms and UMLS semantic types for new hypothesis generation.

Other researches arrange the specific context in order to infer the new relationships between biological objects [8,9]. Srinivasan [8] suggested novel uses of dietary and pharmacological substance in terms of the Swanson’s ABC model. They identified that some diseases were related with curcumin. In the Swanson’s ABC model, they selected context, curcumin as the A terms in an open discovery procedure. The B, C terms were extracted by MeSH terms from the results of searching A term, curcumin in the PubMed. Patric [9] developed the literature mining method called RaJoLink to discover hidden relations by the Swansons’s ABC model in the autism domain.

The major concerns with the ABC model are that 1) it does not incorporate context information into relation inference; 2) it generates a large volume of false positive candidate relations; and 3) it is a semi-automatic, labor-intensive technique requiring human experts' manual input. The goal of this study is to infer unknown interactions among entities extracted from the literature of a particular disease by making use of context term patterns. The proposed approach is particularly useful for drug discovery that determines the possibility of drug repositioning.

Context terms, in this paper, are defined as biological entities co-occurring with interaction pairs in a given PubMed record. These biomedical terms can be treated as context terms of the interactions. The major hypothesis explored in this paper is that the similar context terms co-occurring with interactions among entities provide meaningful patterns to detect undiscovered knowledge.

To our best knowledge, none of previous studies resembles our approach that detects context term patterns from text and applies them for the ABC model problem. The most similar one is by Baker and Hemminger [10] proposing a mining technique based on term co-occurrence based relation model to generate new hypotheses of drug-disease, gene-disease, and drug-function relations. This approach uses diseases-genes-drugs patterns. However, their approach did not utilize context information where interaction of entities is discovered.

We have developed a methodology to extract terms which refer to biological objects from biomedical literature and store them in a repository used to identify interaction pairs of entities from multi-level interaction databases. Applying Swanson's ABC model to stored interaction data, we produced new possible interaction candidates. By applying context term patterns for the interaction results, we were able to improve the precision rates than previous ABC methods.

The contributions of our study are three-folds: First, we built a multi-level interaction database used to identify whether interaction exists among extracted entities. Second, besides the interaction identified by the multi-level interaction DB, we detected patterns of neighbor entities, built pattern vectors, and inferred undiscovered interaction. Third, we validated our approach with 77,711 PubMed records of Alzheimer's disease.

The rest of the paper is organized in the following order. Datasets and methods describe our approach: data collections, evaluation strategy, and evaluation measure. Results and discussions report the experimental results and discuss the results. In conclusions, we conclude the paper with the future plan.

## Datasets and methods

In this section, we describe our approach to hidden interaction extraction using multi-level context terms. First, we describe the dataset used in this paper. Second, we introduce the multi-level entity recognition method as well as the interaction extraction method. Third, we explain the concept of context terms and the context similarity-based scoring method. Last, we describe how we evaluated our method.

### Dataset

#### UMLS

We use UMLS (Universal Medical Language System) [11] to find bio-medical entities from the text. As UMLS is composed of many different thesauruses, we applied different export criteria before importing them to our databases. We first chose the NCI thesaurus as our main thesaurus, because NCI thesaurus accurately categorized drug, disease, and symptom entities. Since the UMLS data is composed of the hierarchical structure, from certain nodes, all descendant nodes are considered to be in the same category. We can use periods (,) to represent hierarchical structures in UMLS. For example, a hierarchical structure, 'A0001.A0002', means that 'A0002' entity is under 'A0001' entity category. We searched for each category entities of gene, disease, and symptom in UMLS. As the result, the entities under 'A1412976.A7570735' can be treated as gene entities, the entities under 'A1412976.A7644030.A12793852' can be treated as disease entities, and the entities under 'A1412976.A7644030.A7580815.A7612336.A7589770' can be treated as symptom entities. After we locate entities, we collect all synonyms by extracting all entities which have the same CUI in the UMLS database.

We extracted 96,031 disease synonyms, 45,527 gene synonyms, and 6,132 symptom synonyms from UMLS in total. Using these synonyms, we built up multi-level biomedical synonym databases of UMLS, and tagged biomedical terms in literatures as context terms.

#### PharmGKB

We use PharmGKB (Pharmacogenetics Knowledge Base) [12] as a dictionary and the golden standard of this study. As a dictionary, we use names of genes, diseases, and drugs in PharmGKB to extract drug-gene or gene-disease relationships. As an answer set, we use interaction pairs between drug and disease defined in PharmGKB.

We extracted 25,693 disease synonyms, 28,091 drug synonyms, and 258,840 gene synonyms from PharmGKB in total. Using these synonyms, we created multi-level biomedical synonym databases out of PharmGKB, and tagged biomedical terms in PubMed records as interaction entities. Also, we extracted 1,992 drug-disease interaction pairs from PharmGKB, and used them as an answer set for the evaluations.

#### CTD

We use CTD (Comparative Toxicogenomics Database) [13] for tagging chemicals, genes, diseases entities in the text. CTD contains 384,141 chemical synonyms, 679,701 gene synonyms, and 68,211 disease synonyms. CTD provides us for an answer set of interactions between diseases and drugs, whose number of interactions is 336,693.

#### PubMed

PubMed is a well-known biomedical literature repository that is widely used for text mining. PubMed has more than 20,000,000 papers' abstracts. For the evaluation, we downloaded 77,711 abstracts of Alzheimer disease in the XML form.

## Methods

**Figure 2:**
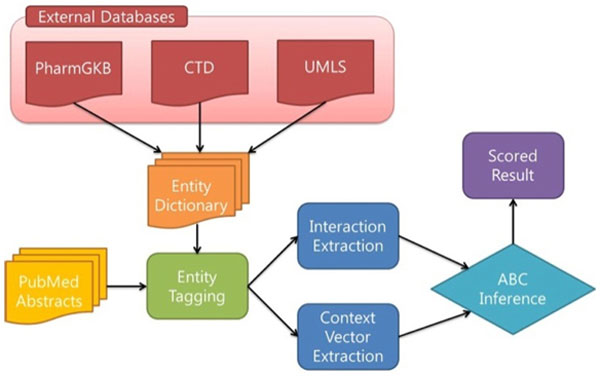
**Overview of our approach**.

In this section, we describe the method to detect undiscovered interactions from the literature using context terms, and we also explain the evaluation strategy and measures. Figure 2 shows the flow diagram of our approach. First, we constructed multi-level entity dictionaries from three external databases (PharmGKB, CTD, and UMLS) and extracted entities from the abstracts. Second, we extracted interactions from the entity set with context term vectors. Third, we inferred undiscovered interactions from the known interactions using context vectors. Finally, we evaluated our results by comparing with the frequency based ABC model.

### Multi-level entity recognition

The main purpose of the entity dictionary databases is to tag multi-level biomedical entities existing in PubMed records. We use 4 levels of entities such as genes, drugs, diseases, and symptoms. To this end, we first parse the sentences using the Conditional Random Field (CRF)-based sentence detector. Second, we match the extracted entities with PharmGKB, and CTD entity dictionary databases to extract interaction data. And third, we map them to the UMLS entity dictionary database to extract context vectors.

We recognize multi-level entity terms from the PubMed records. Prior to entities tagging, we construct a multi-level entity dictionary to recognize each level terms.

**Figure 3:**
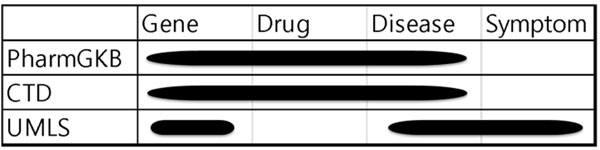
**Entity levels and databases from which the entities imported**.

We import data from three external databases to generate the multi-level entity dictionaries: PharmGKB, CTD, and UMLS (see Figure 3). We first define the entity levels of the dictionaries into four different levels: gene, drug, disease, and symptom. From PharmGKB and CTD databases, gene, drug, and disease entities are imported. From UMLS database, gene, disease, and symptom entities are imported.

Entity dictionary tables are structured as two fields: term names and accession IDs. The content data are imported from each database. We extract names and synonym terms with their accession IDs. Due to this, there can be same accession IDs with different terms in the dictionary databases.

### Interaction extraction

To extract interactions from the identified multi-level entities, we use two kinds of dictionaries such as PharmGKB and CTD. From these, we create dictionary databases. Gene, drug, and disease entities are tagged, and these serve as the candidate entities for candidate interactions.

To extract biologically meaningful interactions, we limited extracted patterns to 'drug - gene' and 'gene - disease' from the recognized entities. These three patterns (A: Drugs, B: Genes, C: Diseases) based AC inferences enable us to find novel relationships between drugs and diseases which can be also called drug repositioning. We can find new drug target diseases using this method, which are inferred by connecting genes.

As shown in Figure 2, we generate entity dictionaries from PharmGKB and CTD databases. We only use names of drugs (chemicals), genes and diseases in these two databases. Based on the names, we tagged biological entities from PubMed records. After we tagged them, we extracted candidate interactions when two different types of entities co-occur within a sentence.

Based on the dictionary database, we obtain different results. PharmGKB and CTD databases have different synonym terms, so their tagging results are different from each other.

### Context vector based similarity scores

To discover the meaningful relationships between AB and BC interactions, we utilize context vectors. Context vectors are defined as the term frequency vectors of each interaction. After extracting interactions, multi-level biomedical entities that were previously extracted and tagged are members of context vectors. In a given PubMed collection, the same interaction can occur in many records, and context terms may be aggregated for one interaction. The average occurrence frequency for each context terms is defined to be a context term pattern for the interaction.

**Figure 4:**
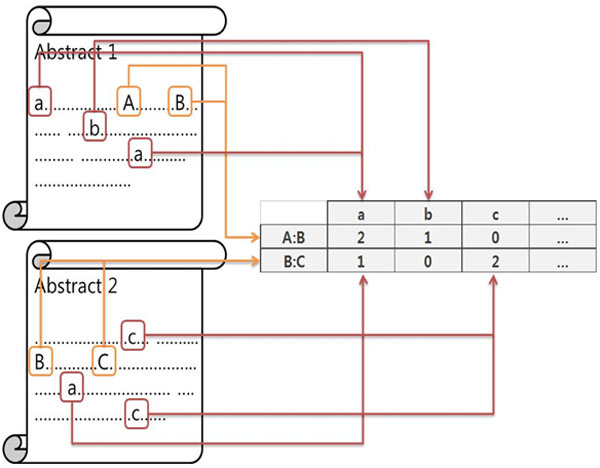
**Example of context vectors for similarity scores calculation**.

Figure 4 shows an example of a context vector. Context vectors contain all biomedical entities tagged from a set of PubMed records of a particular disease. We generate context vectors with the frequency of each term for each interaction. In Figure 4, for example, the "Abstract1" has A:B interaction and "a, b, a" terms occur together. Therefore, the number of counts of "a" term is 2 and the number of counts of "b" term is 1. The "Abstract2" case is the almost same as the "Abstract1" case. Interaction B:C occurs in the "Abstract2" and terms "a, c, c" occur together in the abstract level. Therefore, the number of counts of "a" term is 1 and the number of counts of "c" term is 2. Interaction can be occurred in several abstracts. In this case, we calculate average frequency scores of context vectors as follows:

$$\text{CVi = }\frac{\sum_{j=1}^{n}CVij}{n}$$

where we define CV_i _that denotes a Context Vector for i_th _interaction. We set j as j_th _abstract and n as the number of abstracts includes i_th _interaction.

After construction of CV vectors of each interaction, we calculate similarity scores using both cosine similarity and Spearman correlation. Cosine similarity is widely used in text mining as a measure of similarity between two vectors.

Cosine similarity $\left(\text{C}{{\text{V}}_{\text{A}}}_{,}\;\text{C}{\text{V}}_{\text{B}}\right)=\frac{\sum_{i=1}^{n}CV_{Ai}*CV_{Bi}}{\sqrt{\sum_{i=1}^{n}{\left(CV_{Ai}\right)}^{2}}*\sqrt{\sum_{i=1}^{n}{\left(CV_{Bi}\right)}^{2}}}$

Spearman correlation [14] is also widely used in text mining as a measure of similarity between two vectors.

Spearman Correlation $\left(\text{C}{\text{V}}_{\text{A,}}\;\text{C}{\text{V}}_{\text{B}}\right)=1-\frac{6*\sum_{i=1}^{n}\left(CV_{Ai-}CV_{Bi}\right)}{n\left(n^{2}-1\right)}$

Besides cosine similarity and Spearman correlation measures, we have tried various different similarity measures including Pearson correlation, Jaccard index, RBFKernel, and we found that Cosine and Spearman made the steady, superior performance over the other measures. Thus, we only reported the results by cosine similarity and Spearman correlation measures in this paper.

### Multi-level interaction database

To generate the golden standard answer set, we created multi-level interaction databases. From PharmGKB, and CTD databases, we extracted interactions to create the multi-level interaction database. The database is composed of two accession IDs of interaction entities. Among extracted interactions, we used drug-disease interactions, because our inferred results are limited to only drug-disease interactions. We extracted 1,992 interactions from PharmGKB database, and 336,693 interactions from CTD database.

### Evaluation method

The A-C (Drug-Disease) interactions inferred using B (gene) terms are evaluated by examining how many inferred interactions are matched in well-known interaction databases such as PharmGKB and CTD. We compare our method to the ABC model that is based on entity frequency in Alzheimer's disease related abstracts.

We retrieved a set of PubMed records with an "Alzheimer's disease" as a query. We downloaded 77,711 Alzheimer's disease related papers from the PubMed. Each record is further parsed into sentences by the Conditional Random Field (CRF) technique [15]. We used the Java-version of CRF implemented in the Stanford NLP package [16]. To extract the biological entities from sentences, we use the LingPipe's Hidden Markov Model (HMM)-based technique [17]. For our study, we utilize the NER model trained on the Genia corpus [18]. The extracted named entities are biomedical concepts in a sentence such as "*5 and 10 lM parthenolide", "endoscopy"*, or *"myocardial infarction"*. To ensure that the identified named entities correspond to a controlled set of vocabulary, we map them to concepts from the UMLS database.

**Figure 5:**
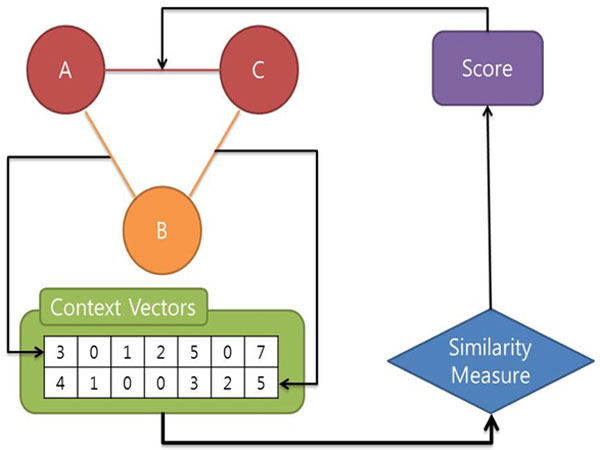
**Calculation of similarity scores for the evaluation**.

Figure 5 shows how the calculation of similarity scores is done for the evaluation. As we infer a new interaction 'A-C' from 'A-B', and 'B-C', we calculate the similarity score of 'A-C' depending on 'A-B', and 'B-C' interactions. First, we build context vectors for each interaction. As they have the same dimension (the number of attributes), we can calculate similarity measures between two vectors. We use both cosine similarity and Spearman correlation as our similarity measure. If the cosine similarity value exceeds a certain threshold, we regard the inference is meaningful. As 'A-C' can be inferred from various 'B' terms, we collect several 'B' terms with cosine similarity scores over the threshold. And then, we calculate the frequency of the inference by multiplying the frequency of 'A-B' interaction with the frequency of 'B-C' interaction.

For every meaningful 'B' terms, we apply three different techniques utilizing frequencies for inference to get a similarity score for 'A-C' interaction. The first method is the sum score. For this score we calculate just summation all scores of A-B_1_-C to A-B_n_-C Pairs. The second is the max score. For this score we use the maximum score of A-B_1_-C to A-B_n_-C. The third one is the hybrid score. For this score we utilize both similarity and frequency of interaction information. We sum up the frequency of interactions when similarity scores of them are greater than a pre-defined threshold.

## Results and discussion

In this section, we report the experimental results by comparing the frequency based ABC model (baseline) and our context terms based model. We evaluate our method with two answer sets, PharmGKB and CTD. We infer interactions related with "Alzheimer's disease" for the evaluation.

### Results of tagging and extraction

In PubMed, we found 77,711 abstracts related with "Alzheimer's disease". There were 1,640,761 biomedical entities in them extracted by our text preprocessing technique. 295,419 of them were tagged by the PharmGKB entity dictionary, 438,987 of them were tagged by the CTD entity dictionary, and 260,291 of them were tagged by the UMLS entity dictionary. We generated 12,432 interactions using PharmGKB tagged entities, and 84,286 interactions using CTD tagged entities. As a result, the size of the context vector for each interaction was 1,641. We generated 14,481 new disease-drug interactions using PharmGKB, and 136,570 interactions using CTD.

### Evaluation

In the evaluation, we focused on how many interactions were matched with two answer sets (known interaction databases - PharmGKB and CTD) as the performance evaluation. For the comparison, we used the frequency based ABC model as the baseline.

**Figure 6:**
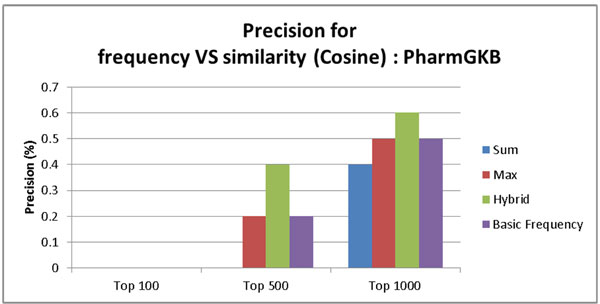
**Precision rate for the baseline ABC model based on frequency count, similarity score summation, max similarity score, and hybrid score on PharmGKB using cosine based similarity**.

**Figure 7:**
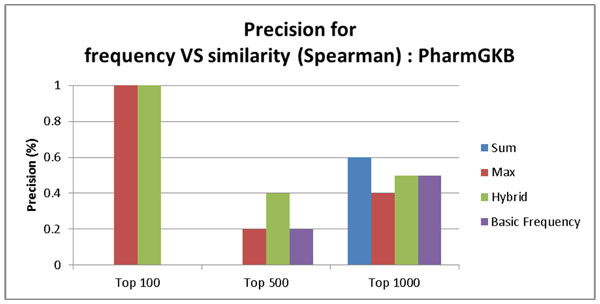
**Precision rate for the baseline ABC model based on frequency count, similarity score summation, max similarity score, and hybrid score on PharmGKB using Spearman correlation based similarity**.

**Figure 8:**
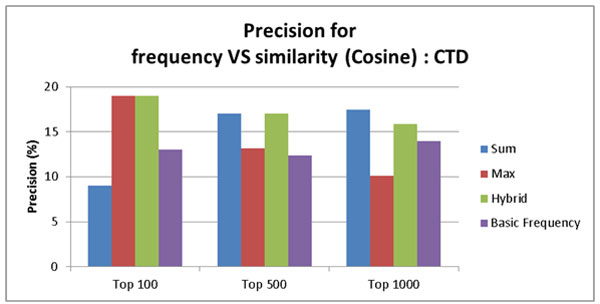
**Precision rate for the baseline ABC model based on frequency count, similarity score summation, max similarity score, and hybrid score on CTD using cosine based similarity**.

**Figure 9:**
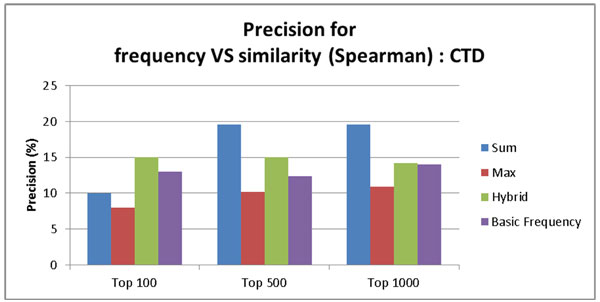
**Precision rate for the baseline ABC model based on frequency count, similarity score summation, max similarity score, and hybrid score on CTD using Spearman correlation based similarity**.

Figure 6 and 7 show precision of the ABC model based on the frequency count and three similarity-based models (Sum, Max, and Hybrid) on PharmGKB. The comparison was made for top 100, top 500, and top 1000 pairs. Figure 6 shows the result using the cosine similarity measure. Figure 7 shows the result using the Spearman correlation similarity measure. The PharmGKB case (Figure 6 and 7) shows that all four approaches based on both cosine similarity and Spearman correlation do not achieve the outstanding performance (between 0%~1%). The weak performance is attributed to the fact that PharmGKB has only 1,992 drug-disease interactions. Furthermore our dataset was not all PubMed abstracts but only Alzheimer's disease related context. In the CTD case (Figure 8 and 9), the results show the better precision performance (~19%), and the reason for the superior performance is because as an answer set CTD had many interactions (336,693 interactions).

Our context similarity based method (Hybrid) is superior to the frequency based ABC model in all cases (Top 100, 500, and 1000) on PharmGKB. In this case, the Hybrid approach sets the threshold for cosine similarity to 0.945 and for Spearman coefficient to 0.999999999.

Figures 8 and 9 show the performance results according to the precision by the ABC model based on the frequency count and similarity based models (Sum, Max, and Hybrid) on CTD. The comparison was made for top 100, top 500, and top 1000 pairs. Figure 8 shows the results using cosine similarity measure. Figure 9 shows the results using Spearman correlation. For the CTD case, only the hybrid approach is superior to the baseline in all cases (Top 100, 500, and 1000) using the threshold 0.95 for cosine similarity and 0.999999998 for Spearman correlation. This result indicates that the frequency of interaction is an important factor to find meaningful relationships because only the hybrid method incorporates both frequency and similarity information. These results also show that the context term based similarity is a good feature to find meaningful relationships. When we filtered the inferred interactions using the context term based similarity, we observed that it helped improve performance, which is better than the frequency used only.

### Literature analysis of top 10 interactions

In this section, we analyzed top 10 ranked results to examine whether high ranked interactions are truly meaningful or not. The answer set of CTD contains not only known interactions reported in the literature but also interactions based on the related genes that are undiscovered and not reported in the literature. To examine whether high ranked interactions are truly meaningful or not, we searched literatures for the known evidences. For the comparison, we analyzed the top 10 ranked results of the cosine similarity based hybrid method and the frequency based ABC method.

Table 1 shows that top 10 ranked interactions from the cosine based hybrid approach using CTD entities information. According to the literature analysis, we found that 7 interactions are meaningful among the top 10 interactions using context based approach. D058225:D016229 is the top ranked interaction which is not in the CTD databases. We found that Amyloid beta-peptides are the principal component of amyloid plaques from the literature [19]. We also found that the other top 4 ranked interactions are not in the CTD databases but in the literatures. Up-regulating Nerve Growth Factor (NGF) produced therapeutic effect on AD in rats [20].

**Table 1:** Top 10 ranked interactions from the hybrid approach (cosine)

CTD-complex0.95	Disease	Chemical	PMID
D058225:D016229	Plaque, amyloid	Amyloid beta-peptides	21575663
D000544:D020932	Alzheimer disease	Nerve growth factor	20965859
D005182:D000544	Alzheimer disease	Flavin-adenine dinucleotide	12127087
D015850:D000544	Alzheimer disease	Interleukin-6	20667498
D000544:D014409	Alzheimer disease	Tumor necrosis factor-alpha	21327054
D000544:D015415	Alzheimer disease	Biological markers	
D005182:D002311	Cardiomyopathy, dilated	Flavin-adenine dinucleotide	
D000544:D016229	Alzheimer disease	Amyloid beta-peptides	21726674
D002311:D016229	Cardiomyopathy, dilated	Amyloid beta-peptides	
**D000544:D007328**	**Alzheimer** **disease**	**Insulin**	**21525299**

In the case of D005182:D000544, we found that flavin-adenine dinucleotide dependent oxidoreductases are shared similarities with seladin-1 gene and down-regulated seladin-1 in brain regions selectively degenerated in AD from the literature [21]. Interleukin-6 has relations on AD also reported in the literature [22].

Tumor necrosis factor (TNF) alpha has also relations on Alzheimer disease [23]. Amyloid beta-peptides are known as a main component of senile plaques (SPs), and senile plaques is highly related to Alzheimer's disease [24]. Finally, Alzheimer disease has a similar risk factor with type 2 diabetes and has relations with insulin reported in this literature [25].

Table 2 shows that top 10 ranked interactions from the frequency based ABC model using CTD entities information. These results are almost identical with the results reported in Table 1. These results in Table 2 show that the top 8 of 10 interactions are same between the cosine similarity-based hybrid approach and the frequency based ABC model. We found that the top 5 ranked interactions were confirmed in literatures. We also found that the 8th ranked interaction also had the evidence on the literature. The 9th ranked and 10th ranked ones were different between the hybrid and the frequency based approach. As shown in the 10th ranked interaction, only our hybrid approach was able to infer the relationship unlike the basic ABC model failing to infer it in the top 10 ranked interactions. Our hypothesis was that the similarity of context terms between A-B and B-C model enables to infer more meaningful interactions A-C. Literature analysis confirms that in the candidate interactions inferred by the ABC model, the similarity based hybrid approach penalizes less meaningful interactions by ranking them.

**Table 2:** Top 10 ranked interactions from frequency based basic ABC model

CTD-Basic ABC	Disease	Chemical	PMID
D058225:D016229	Plaque, amyloid	Amyloid beta-peptides	21575663
D000544:D020932	Alzheimer disease	Nerve growth factor	20965859
D005182:D000544	Alzheimer disease	Flavin-adenine dinucleotide	12127087
D015850:D000544	Alzheimer disease	Interleukin-6	20667498
D000544:D014409	Alzheimer disease	Tumor necrosis factor-alpha	21327054
D000544:D015415	Alzheimer disease	Biological markers	
D005182:D002311	Cardiomyopathy, dilated	Flavin-adenine dinucleotide	
D000544:D016229	Alzheimer disease	Amyloid beta-peptides	21726674
607842:D016229	Aural atresia, congenital	Amyloid beta-peptides	
D000544:D014212	Alzheimer disease	Tretinoin	

## Conclusions

In this paper, we proposed a novel approach to infer undiscovered interactions from the literature. We defined interaction specific context vectors which model a specific condition of interactions. We inferred interactions from the known A-B interactions in the literature by utilizing the context vector similarity. We presented three different context vector similarity based scoring functions (sum, max, hybrid). We evaluated our method using PharmGKB and CTD. The results show that the hybrid approach constantly performs better than the frequency based ABC model in all cases. The literature based analysis of top 10 ranked interactions confirms that our hybrid based approach could find more meaningful interactions than the frequency based ABC model.

In the future study, we plan to improve the precision of this method. We believe that the size of the context vector is very large and sparse because we used all bio-medical entities which were tagged by UMLS. If we could extract meaningful features from members of the context vector, we will be able to improve the result. We also plan to find hidden interactions that are associated with specific conditions using context vectors. If we could extract context vectors from the known interaction in the specific disease dataset, we will be able to infer the interaction using context vectors of the condition specific interaction. These will enable us to find the novel interaction between drugs and diseases, called drug repositioning.

## Competing interests

The authors declare that they have no competing interests.

## Authors' contributions

LS and JC designed the method and drafted the manuscript along with MS. KP carried out literature analysis for the validation. MS also critically revised the manuscript for important intellectual context and developed the text mining component. DL supervised the work and gave final approval of the version of the manuscript to be submitted.
